# Body Mass and Income: Gender and Occupational Differences

**DOI:** 10.3390/ijerph18189599

**Published:** 2021-09-12

**Authors:** Ping Li, Xiaozhou Chen, Qi Yao

**Affiliations:** 1 School of Economics and Management, South China Normal University, Guangzhou 510006, China; li_ping@m.scnu.edu.cn (P.L.); 2019020309@m.scnu.edu.cn (Q.Y.); 2 School of Business Administration, Guangdong University of Finance, Guangzhou 510521, China

**Keywords:** BMI, overweight, underweight, obesity penalty, weight premium

## Abstract

This paper aims to examine the influence of body shape on income, which varies with gender and occupational structure in China. The data were obtained from the CGSS (Chinese General Social Survey) 2010–2017 Survey. The overall finding in this paper is that women and men face different body shape–income effects. For females, the obesity penalty is significant and is reinforced with increasing occupational rank. For men, the thinness penalty (or weight premium) is enhanced as the occupational class decreases. Body shape–income gaps are mainly caused by the occupational structure. Twenty-nine percent of the income gap between overweight and average weight women can be explained by the obesity penalty, 37% of the income gap between overweight and average weight men can be interpreted by the weight premium, and 11% of the gap between underweight and normal weight men can be explained by the thinness penalty. The findings also suggest that the effect of body shape on income consists of two pathways: body shape affects health capital and socialization, and therefore income. Healthy lifestyles and scientific employment concepts should be promoted, and measures to close the gender gap should be implemented.

## 1. Introduction

### 1.1. Background

According to the WHO (World Health Organization), the worldwide prevalence of obesity, defined by BMI (Body Mass Index) of over 30 kg/m^2^, nearly tripled since 1975, and 39% of adults aged 18 years and older were overweight or obese in 2016 (World Health Organization. Obesity and overweight, 2018. Available: https://www.who.int/news-room/fact-sheets/detail/obesity-and-overweight (accessed on 9 June 2021)). Body shape is the dominant factor that influences others’ first impressions of individuals. The labor market feedback on body shape is embodied in the income gap premium and penalty of body shape. There is mounting evidence that higher rates of obesity reduce labor market attachment, worker productivity, and earnings [1,2,3,4,5].

The relationship between body size and labor outcomes was studied based on data from different countries: United States [6,7,8], European [9], Germany [10], British [11], Danish [12], Spanish [13], Philippines [14], and China [15], etc. Results could differ based on the sample and data. Whether the premium and penalty from body shape are high or low, it already affects the labor outcome of the worker at the micro-level and has also already affected the economic growth at the macro-level [16].

### 1.2. Literature Review

The economics literature focuses on the feedback mechanisms of body shape in the labor market, such as the slim premium and the obesity penalty. The main reasons for the income gap of body shape include the following.

First, obese individuals face employer and customer discrimination. Both employers and customers have stereotypes of obese people, such as frequent absenteeism, low productivity, laziness, lack of self-control, lack of willpower, etc. [17,18]. Obese individuals are at a significant disadvantage in employment, access to training opportunities, wages, and job promotions due to employer discrimination [19]. Hiring managers holding more negative automatic stereotypes about the obese were less likely to invite an obese applicant for an interview. Automatic bias predicts labor market discrimination against obese individuals [20]. Practical implications are discussed. Persons who are obese are unlikely to be promoted to core positions and accordingly receive lower wages [13]. 

Second, body shape affects earnings through the transmission channels of self-esteem. Stereotypes directly undermine workers’ self-esteem and confidence, adding to the psychological burden of obese individuals. BMI was associated with different perceptions of body weight [21]. Since obesity leads to lower self-worth perceptions, obese workers voluntarily exchange more inadequate wage compensation for greater employment possibilities [6]. On the other side, physically attractive workers are higher in confidence, which increases wages. For a given confidence level, physically attractive workers are (wrongly) considered more able by employers [22,23].

Third, body shape is often associated with health status and, therefore, productivity. Obesity is a risk factor for various diseases, and an increased risk of disease among employees can lead to increased health insurance costs for employers. Health-related factors and obese workers’ behavior patterns may be the channels through which obesity adversely affects wages [24,25]. Zhang, et al. used Chinese data to examine the relationship between body shape and health and found that obese men and women were 35.7 and 34.6% more likely to be sick than normal weight workers, respectively [15]. With or without discrimination against obese people, for total compensation to remain stable, employers must balance more abundant health benefits with lower wages and therefore are likely to pay obese or excessively thin people lower wages [26]. Moreover, overweight people know that obesity is a risk factor for various diseases and are more inclined to choose jobs that offer rich health benefits, such as health insurance, pension insurance, and life insurance, at lower wage levels [27].

Health capital often directly affects the physical strength, work capacity, and productivity of workers. Kinge used dynamic models to indicate an inverse u-shaped association between BMI and wages. Other things being equal, obese people may be less productive than nonobese people and therefore less likely to earn higher wages [2]. A BMI that is too high implies an increased health risk for the individual, and a physical capacity that is too low then leads to low fitness and low productivity of the worker [28]. Lost productivity on the job is often the result of health problems and subsequent injuries, and a lack of control over the pace and schedule of work. This can contribute substantially to the indirect costs of worker health problems [29]. 

There is also more research focusing on gender gaps of body shape affecting employment and wages [9,24,30,31,32]. Males suffer more from shortness penalties, whereas females may benefit from pretty faces [33]. Due to the inherent gender discrimination against women in the labor market, women are more likely to experience employer and customer discrimination than men if they are overweight or underweight [17]. By comparing employer discrimination against obese workers for men and women, some studies [34,35] found that women are at greater risk for weight/height discrimination than men, especially women with a BMI of 30–35. The latter were three times more likely to report weight/height discrimination compared to male peers of similar weight. Weight loss positively affects the employment prospects of obese women but not of obese men [23]. Women suffer lower income due to being overweight, but overweight men have higher income. Judge, et al. [36] found a negative weight–income relationship is steepest at the thin end of the distribution for women. There is a positive weight–income relationship across the weight distribution until obesity, where it becomes negative for men. Greve found an inverted u-shaped effect for men, because overweight men can be muscular with little body fat [12].

A study in South Korea, an Asian country, found that obese and overweight men were 1.46 times more likely to be placed in professional jobs and had 13.9% higher monthly wages than their normal-weight counterparts. However, obese and overweight women were 0.33 times less likely to have service jobs, earned 9.0% lower monthly wages, and were half as likely to have jobs with bonuses as their normal-weight counterparts [37]. Sabia and Rees found that overweight and obese white women face a wage gap of 15% less than normal weight women [8].

The gender gap in the body shape effect arises largely due to the occupational structure distribution. Obesity rates and wage penalties for being overweight or obese may vary by occupation [7,13,38,39]. More women are in service and clerical occupations, while a greater percentage of men are in blue-collar occupations. There are still substantial gender gaps in reservation wage and employment due to gender stereotypes, health factors, household, and childcare [40,41,42]. Although the occupational structure has changed dramatically due to increased female participation in the labor market, gender employment differences persist [43,44]. Wage penalties for overweight and obese women are only observable in white-collar occupations [45]. Obese workers may be more vulnerable to wage penalties in occupations with a high frequency of customer interactions. In customer-oriented service and sales jobs, there are often more stringent requirements for employee body shape. The association between BMI and income is stronger for occupations that require more interpersonal skills [13,46]. Subtle increases in BMI within a healthy weight range still reduce women’s employment chances in the service sector [47].

### 1.3. Study Outline

This paper investigates the relationship between body shape and income in China to better understand the mechanisms by which body shape affects earnings. We consider the differences in weight across occupations and therefore analyze the interaction term between weight and occupation. It explores the obesity penalty for women and the weight premium for men under the influence of different occupational classes. Additionally, the data are more recent than those in earlier Chinese studies, as recent survey data from 2017 were included.

We start our analysis in this paper by estimating a series of models that compare male and female body shape premiums and penalties, using interaction terms to identify differences between occupations. Then, we analyzed the occupational heterogeneity of body shape effects and decomposed the income gap to figure out how much body shape premium or penalty accounted. At last, we confirmed the pathway of the impact of body shape on income in terms of both health capital and socialization. 

## 2. Material and Methods

### 2.1. Data

The data for empirical analysis of this article came from the CGSS. The Chinese General Social Survey is a nationwide, comprehensive, and continuous academic survey project in China. Thus far, two phases have been carried out. The first phase was during 2003–2008, in which 5 annual surveys were completed, and the second phase was during 2010–2019, in which 6 annual surveys were completed in 2010, 2011, 2012, 2013, 2015, and 2017. The data used in this article are the survey data from the second phase, including data from 2010 to 2017. The combined data were used for empirical analysis. The survey was completed each summer. The sampling schemes used for the survey were over 100 county units plus 5 metropolitan areas, with a sample of approximately 10,000–12,000 individual respondents per year. However, the original sample size in 2011 was only 5620. We have included year fixed effects in all equations to avoid measurement bias due to the sampling schemes by year.

The CGSS data include the height and weight of the respondent, occupational information, individual income, labor income, education, and other individual variables, which provide good data support for analysis of the income gap. When processing the data: (1) we limited the sample to 18–60 years old workers. The legal working age in China is 16–60 years old. Below 18 years old are underage workers, and the law restricts the occupations they can work. To avoid the impact of external regulations on occupational influence, we remove workers under 18 years old, although they represent a small percentage; (2) we omitted subjects with missing BMI values and retained subjects with BMI between 10 and 40 to ensure that excessively obese or excessively thin samples were not included; (3) this paper is based on a study of the working class, which makes up most of the population. Respondents earning more than CNY 1 million accounted for less than 1% of the valid sample. Most of them were not employed but self-employed or senior professional managers, and more often than not, they were the perpetrators rather than the sufferers of body shape discrimination. To avoid extreme values, we adjusted income over CNY 1,000,000 to CNY 1,000,000, although this applied to few observations; (4) soldiers and farmers are both very special occupations, and we excluded military and farmer subjects, allowing us to obtain a civilian, nonagricultural labor market sample in China; (5) we merged the mixed multi-period survey data with macro data to further obtain the economic development and health care conditions in the individual’s province, facilitating the use of instrumental variables and controls for regional effects. The sample size after data processing was 30,146, with 14,467 women and 15,679 men, and the gender composition was basically balanced.

Two aspects determine the size of an individual: height and weight. In the same weight state, individuals of different heights present other physical characteristics. The most common indicator used to measure body shape is body mass index (BMI), BMI = weight/height^2^, where weight is in kilograms and height is in meters. Many studies use BMI directly to measure body shape [10,48,49]. The classification of absolute BMI values by WHO and countries varies according to national ethnography. In general, those between the BMI 30 and 70% quartiles are often defined in economics and sociological studies as having a normal body shape, those in the high 30% quartile of BMI are considered overweight, and those in the low 30% quartile of BMI are considered underweight. In this paper, we follow this practice and define three types of body shapes after sorting the male and female samples separately. We also used a 20 and 80% partitioning approach at the end, which had little impact on the conclusions. The division of the body shape categories is shown in Table 1.

This paper does not consider the appearance factor because, first, the judgment of appearance by interviewers and respondents is highly subjective and influenced by personal aesthetic preferences. Second, appearance is influenced by income and occupation. Men’s hair arrangement and women’s makeup will obviously change the intuitive feeling of appearance, closely related to individuals’ income level and the nature of their occupation; moreover, suitable instrumental variables are not easily found. To avoid endogenous problems, we consider body shape as a personal appearance characteristic only. We also assume that body shape is not affected by income in the short term and that workers do not gain and lose weight due to sudden increases and decreases in income levels. Body shape is an exogenous factor for the individual’s income equation in the short term. Due to significant differences in physical characteristics between males and females and to avoid the effect of gender discrimination on labor income, we study male subjects separately from female subjects. 

Scholars have looked for instrumental variables to address endogeneity. Instrumental variables must be related to weight but not income. Therefore, some scholars focus on the genetic factors of body shape [50] and thus use the BMI of the relatives of the respondents as an instrumental variable. In addition, variables used as instrumental variables for body shape include the lagged component of the respondents’ own BMI [25], the mean adult BMI at the regional level [30], the obesity status of biological relatives [11], oldest child’s BMI [2], and childhood conditions, lifestyle variables, food expenditure, and household shocks [51]. The instrumental variable we used for BMI was the mean BMI by gender, by type of occupation, and by region. The samples were separated by gender in the first stage, by province in the second stage, and by occupation (according to the first subcategory of ISCO08) in the third stage. The mean BMI values were calculated after the classification. We used the mean BMI of the occupation and province as the individual BMI instrumental variable, which satisfied the correlation condition with the explanatory variables but did not directly affect the individual’s income.

The main control variables used in this paper include demographics and socioeconomic status, which are the most commonly used control variables for study employment [2,13]. We additionally had regional control variables since people’s health and body shape are related to regional medical and health conditions, and employment and occupation are relevant to economics. Fixed effects include time fixed effects and region fixed effects. A selective list of variables used in the regression analysis is provided in Table 1. 

### 2.2. Statistical Analysis

The descriptive statistics in Table 2 show that the mean heights of the three body size groups did not differ much and were approximately 1.6 m for women and 1.7 m for men. The main difference was in weight, with significant differences in mean weight between the overweight and the underweight people. The mean weights of the overweight, normal weight, and underweight women were 66, 56, and 44 kg, respectively, and the mean weights of the three categories of men were 79, 67, and 52 kg, respectively. Weight is the key to discriminating BMI values in three types of body shapes. Height is a variable that is dominated by genetics and has a limited impact on individual body shape. The greater impact on body shape comes from weight, and although weight is also influenced by genetic effects, it is more influenced by acquired personal habits and work features. The mean BMI value for women was 21.57, and for men, it was 22.59.

The income gap of the three body shapes is mainly reflected in the lower income of overweight women. The mean incomes of normal weight and underweight women were CNY 35,000 and CNY 34,000, respectively. The mean income of overweight women was CNY 29,000, which is obviously lower than the income of women of other body shapes. We believe there may be an obesity penalty for female workers. However, the income of male workers with different body shape characteristics is a different story: the average income of men was CNY 46,000. The mean income of overweight men was the highest at CNY 52,000. The lowest was for underweight men, with a mean income of CNY 38,000, while the income of normal weight men was CNY 47,000. Being overweight does not appear to negatively influence men’s income, and underweight men should be anxious because they have significantly lower earnings. There are also notable differences in the occupational structure of men and women, as shown in the bottom half of Table 2. The percentage of women in clerks, services and sales was 45.19%, while the rate in blue-collar jobs (craft, machine operators, elementary occupations) was 28.57%. The occupational structure of men is the opposite of women: the percentage of men in clerks, services and sales was 27.57%, while the rate of those in blue-collar occupations was 44.76%. 

**Figure 1:**
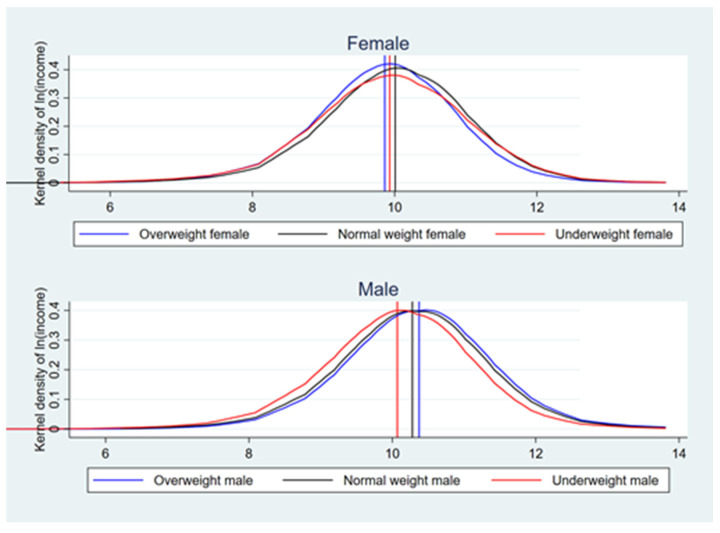
Kernel density of ln (income).

The kernel density plot of the logarithm of income for the three types of body shape (Figure 1) also exhibits the income gap: both the peaks and the mean for normal weight women are on the far right, significantly higher than the logarithm of income for overweight and underweight women. The distribution of log income differs for male workers: overweight males have higher income, with both the peaks and the mean of the log income more to the right, while underweight males have both the peaks and the mean mostly to the left. Based on the distribution of income for males and females, there is heterogeneity in the labor market outcomes for men and women’s body shape. 

## 3. Results

### 3.1. Gender Comparisons of Income Effect

Linear equations are most commonly used for income gap analysis. To provide evidence that body shape indeed affected income, we estimate the following OLS regression:

(1) $$Income=\alpha +\beta_{1}BMI+\beta_{2}Demographics+\beta_{3}Socioeconomic+\beta_{4}Regional+\beta_{5}Fixed+\varepsilon$$

where demographics, socioeconomic status, regional controls, and fixed effects are vectors of control variables. Demographic variables included migration status, race, marital status, logarithm of age, number of children under 18, and education. Socioeconomic status variables included political status, union, medical insurance, and social status. Regional control variables included PGDP, population, number of unemployed individuals, number of benefits, consumption per capital, number of health institutions, number of hospitals, and number of health technicians. Fixed effects included area fixed effects and survey year fixed effects. Provinces were divided into three regions: eastern, central, and western. The logarithm of individual income was used as the dependent variable. We also used labor income as the dependent variable in the robustness test.

The results are reported in Table 3. Panel A shows the regression results for the female sample, and panel B shows the results for the male sample. Models (1)–(3) are OLS estimators. Model (1) is the result of including only BMI and fixed effects; Model (2) is the result of including the control variables demographics and socioeconomic status, and Model (3) is the result of adding regional control variables further.

- The coefficient of the primary variable BMI was significant in both the male and female models, but men and women face inconsistent labor market feedback;
- In the female sample, an increase in BMI implies a decrease in income, as the coefficient is negative (Table 3 Panel A), meaning the obesity penalty that women face;
- In the male sample, the BMI coefficient is positive (Table 3 Panel B), implying that thin men receive less income, meaning the thinness penalty is faced by men.

Considering the effect of sample selection bias, we used Heckman 2SLS as a robustness test, and the results are reported in Model (4). To support the robustness of the findings, the 2SLS with instrumental variables was applied to the estimation. The results are reported in Model (5). Models (4) and (5) demonstrate the robustness of the results quite well.

### 3.2. The Income Effect of Body Size Varies with Occupation

To account for the fact that body shape influence varies among occupations, we added variables for occupation and ISEI and an interaction term of BMI and occupation to the equation to explore whether the same body shape impact exists in all jobs. Using within-group dispersion can reduce the influence of the ISEI and BMI values. The interaction terms added to the equation are all decentralized [52], that is, the ISEI and BMI variable is subtracted from the corresponding mean value. The model used is as follows:

(2) $$\begin{array}{ll} Income= & \alpha +\beta_{1}\left(BMI-\bar{BMI}\right)+\beta_{2}\left(ISEI-\bar{ISEI}\right)+\beta_{3}\left(BMI-\bar{BMI}\right)\left(ISEI-\bar{ISEI}\right)\\ & +\beta_{4}Demographics+\beta_{5}Socioeconomic+\beta_{6}Regional+\beta_{7}Fixed+\varepsilon \end{array}$$

The use of intragroup deviations to estimate coefficients does not have an impact on causality. Therefore, the impact coefficients of BMI are $\left(\beta_{1}+\beta_{3}\Delta ISEI\right)$, which varies with occupation. The regression results are presented in Table 4. Panel A shows the regression results for the female sample, and panel B shows the results for the male sample. Model (6) with demographics and socioeconomic status variables but not for Model (7) with regional controls and fixed effects.

In Table 4 Panel A female sample:

- The coefficient of BMI in Model (7) is −0.008, which differed very little from −0.01 that in Model (3). They were both equations with all control variables added;
- In Models (6) and (7), the coefficient of the interaction term $\beta_{3}$ is significantly negative, implying that the negative effect of BMI on income is strengthened as occupational class increases. That is, the obesity penalty is reinforced in higher class occupations.

In Table 4 Panel B male sample:

- The coefficient of BMI in male Model (7) and Model (3) also differed very little: 0.016 and 0.017, respectively;
- In the male models, the interaction term coefficient is significantly negative for males. The thinness penalty embodied by BMI is more pronounced in low ISEI occupations and is weaker in higher occupational classes.

### 3.3. Occupational Heterogeneity

**Figure 2:**
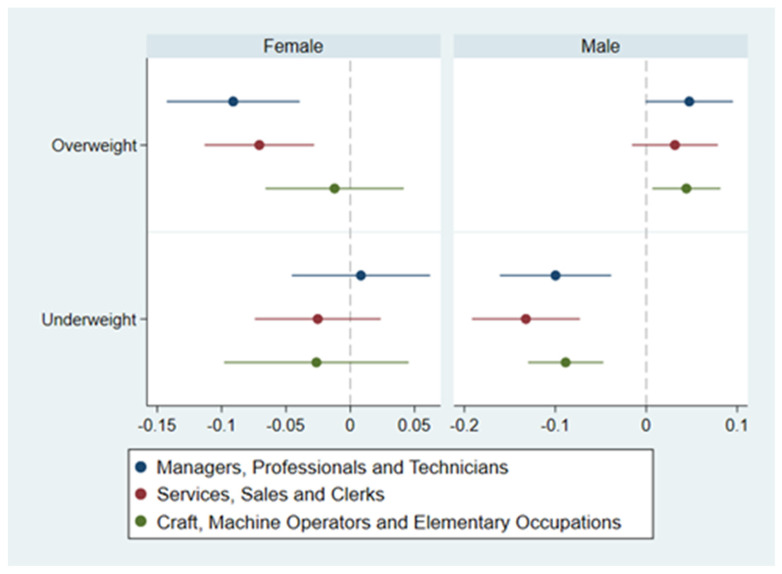
Heterogeneity of the income effect of body shape across occupations. Note: Demographics, socioeconomic status, regional controls, and fixed effects variables were all added in each regression, which had robust standard errors.

We categorized body shape into overweight, normal shape, and underweight, and the regression results for body shape as independent variables by occupations are graphed in Figure 2. We performed separate regressions for three major occupations (The first one was managers, professionals, and technicians; the second one was services, sales, and the third one was clerks and craft, machine operators, and elementary occupations). The coefficients for overweight and underweight individuals are shown in Figure 2.

- In the female sample, the coefficient of overweight was significantly negative in managers, professionals, and technicians and services, sales, and clerks, with a significant obesity penalty. However, in craft, machine operators, and elementary occupations, the obesity penalty was not significant;
- In the male sample, the weight premium was significant in craft, machine operators, elementary occupations, while it was not confirmed in other occupations. The coefficient of underweight was always significantly negative, which means that the thinness penalty was always observed.

### 3.4. Decomposition of Income Gap

**Figure 3:**
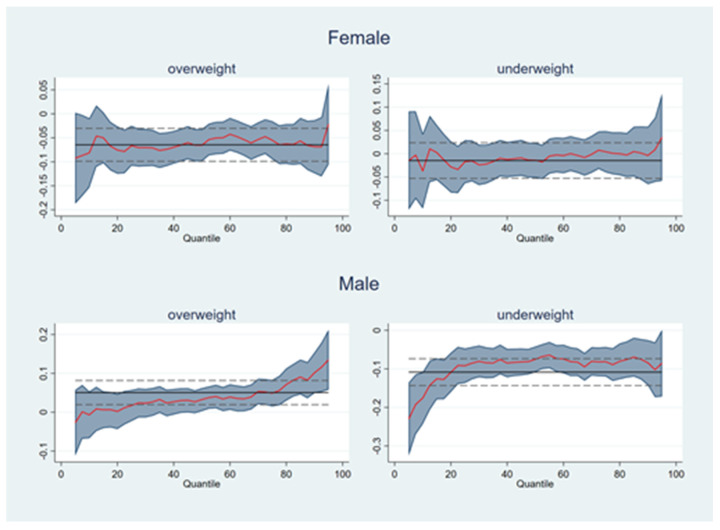
Results of quantile regression. Note: Demographics, socioeconomic status, regional controls, and fixed effects variables were all added in each regression, which had robust standard errors.

The OLS results reflect the effect of body shape on income at the mean income level. Johar and Katayama [53] found significant differences in the effect of appearance characteristics on income at different quartiles. To capture the distribution of body shape and income more accurately and comprehensively, we reanalyzed the income equations for males and females using quantile regression (Figure 3).

- In the female sample, the overweight coefficient was significant in most quartiles and did not differ dramatic across income levels;
- In the male sample, the underweight coefficient was significant in most quartiles. The negative influence of underweight was strongest in the low-income quantile, and then the coefficient values showed an increasing trend, indicating that the thinness penalty is most noticeable in the low-income class. This result can be used as indirect evidence that the income gap changes with occupation.

Based on the income gaps of overweight, underweight, and normal weight, we decompose the income gaps of different body shapes using Brown’s (1980) decomposition method, treating occupation as a mediating variable and considering all control variables to explain the income gap. A linear relationship between income and the explanatory variables is assumed: lninc=Xβ+ε. We obtain the OLS estimation statistics $\hat{\beta }$. The following relationship exists after averaging the two sides of the regression equation: $\bar{\ln inc}=\bar{X}\hat{\beta }$. The income gap can be decomposed into:

(3) $$\begin{array}{ll} {\bar{I}}^{N}-{\bar{I}}^{O} & =\sum_{j=1}^{8}\left(P_{j}^{N}{\bar{I}}_{j}^{N}-P_{j}^{B}{\bar{I}}_{j}^{O}\right)\\ & =\sum_{j=1}^{8}\left[P_{j}^{N}{\bar{X}}_{j}^{N}{\hat{\beta }}_{j}^{N}-P_{j}^{O}{\bar{X}}_{j}^{O}{\hat{\beta }}_{j}^{O}\right]\\ & =\sum_{j=1}^{8}\underset{Part\left(1\right)}{\underline{[P_{j}^{O}{\bar{X}}_{j}^{O}\left({\hat{\beta }}_{j}^{N}-{\hat{\beta }}_{j}^{O}\right)}}+\underset{Part\left(2\right)}{\underline{P_{j}^{O}\left({\bar{X}}_{j}^{N}-{\bar{X}}_{j}^{O}\right){\hat{\beta }}_{j}^{N}}}+\underset{Part\left(3\right)}{\underline{\left(P_{j}^{N}-P_{j}^{O}\right){\bar{I}}_{j}^{N}]}} \end{array}$$

Where superscript *N* is normal weight, superscript *O* is overweight, and ${\bar{I}}^{N}-{\bar{I}}^{O}$ is the income gap between the normal weight group and the overweight group. The subscript j is the occupations, and there are eight occupations. $P_{j}^{N}$ and $P_{j}^{O}$ are the percentages of occupation j in the normal weight women and overweight women. Overall, Part (1) and Part (2) are gaps within occupations, and Part (3) are gaps between occupations. In the composition of the decomposition equation:

- Part (1) is the income gap caused by the variation in regression coefficients, which can be considered as different market feedback from the labor market for the two types of workers: normal weight and overweight women;
- Part (2) is the income gap caused by variation in the independent variable values between the two groups. We estimate OLS lninc=Xβ+ε for each of the eight occupational categories separately, with independent variables including all control variables. Coefficients ${\hat{\beta }}_{j}^{N}$ and ${\hat{\beta }}_{j}^{O}$ were obtained. Part (1) and Part (2) can be calculated;
- Part (3) is the income gap caused by differences in occupational structure. Part (3) of the gap arising from occupational structure is further decomposed to Part (4) and Part (5).

(4) $$\begin{array}{l} {\bar{I}}^{N}-{\bar{I}}^{O}\\ =\sum_{j=1}^{8}\underset{Part\left(1\right)}{\underline{[P_{j}^{O}{\bar{X}}_{j}^{O}\left({\hat{\beta }}_{j}^{N}-{\hat{\beta }}_{j}^{O}\right)}}+\underset{Part\left(2\right)}{\underline{P_{j}^{O}\left({\bar{X}}_{j}^{N}-{\bar{X}}_{j}^{O}\right){\hat{\beta }}_{j}^{N}}}+\underset{Part\left(4\right)}{\underline{\left(P_{j}^{N}-{\hat{P}}_{j}^{O}\right){\bar{I}}_{j}^{N}}}+\underset{Part\left(5\right)}{\underline{\left({\hat{P}}_{j}^{O}-P_{j}^{O}\right){\bar{I}}_{j}^{N}]}} \end{array}$$

The occupational structure difference $\left(P_{j}^{N}-P_{j}^{O}\right)$ is decomposed into $\left(P_{j}^{N}-{\hat{P}}_{j}^{O}\right)$ and $\left({\hat{P}}_{j}^{O}-P_{j}^{O}\right)$, where ${\hat{P}}_{j}^{O}$ is the counterfactual hypothetical occupational structure. What would be the occupational structure of overweight women if they had the same regression coefficients as normal weight women?

We used a multi-logit model for the sample of normal weight women, with the dependent variable being the first major category of ISCO08, and added all control variables to the independent variables. Occupation coefficients were obtained from the regression of normal weight women. Then, the coefficients of the multi-logit model for normal weight women were used to predict the hypothetical occupational structure of overweight women. We obtain the probability of overweight women entering each occupational class if they have the same coefficients as normal-weight women. We obtain the hypothetical occupational structure ${\hat{P}}_{j}^{O}$.

- Part (4) and Part (5) are added to denote the income gap caused by differences in the occupational structure of normal weight women and overweight women. $\left(P_{j}^{N}-{\hat{P}}_{j}^{O}\right)$ represents differences in occupational structure due to control variables, while $\left({\hat{P}}_{j}^{O}-P_{j}^{O}\right)$ represents differences in occupational structure due to occupation regression coefficients.

We can decompose the income gap between normal and overweight men and between normal and underweight men in the same way. The coefficient of underweight was not significant in the female sample. Therefore, we do not decompose the income gap for underweight women. The decomposition results are presented in Table 5. 

Panel A in Table 5 shows the income gap decomposition of normal weight women and overweight women. The gap in the logarithm of income between the normal weight women and overweight women is 0.15, and the gap in Panel A is 0.145, which decomposes 97% of the gap. Part (1) plus Part (2) is the income gap within the occupational category, which accounts for 55%. Of these, 29% of the income gap is due to coefficient variation. That is, the labor market has different feedback for normal weight and overweight women, with normal weight having higher income in most occupations (Table 5 Panel A Part (1)). The income gap explained by variations of control variables accounts for 27% (Table 5 Panel A Part (2)). The gap between occupational categories accounts for 45%, which is due to the different occupational structures between normal weight and overweight women. They may also be treated differently when searching for jobs. The difference in employment structure due to explanatory variables is $\left(P_{j}^{N}-{\hat{P}}_{j}^{O}\right)$, which explains 37% of the income gap (Table 5 Panel A Part (3)). In summary, the difference in income regression coefficients $\left({\hat{\beta }}_{j}^{N}-{\hat{\beta }}_{j}^{O}\right)$ largely confirms the income gap between normal weight and overweight women due to the obesity penalty, which explained 29% of the income gap. 

Panel B in Table 5 shows the income gap decomposition of normal weight and overweight men. The income gap between the two groups is −0.09, which is negative, meaning that overweight men earn more, ${\bar{I}}_{male}{}^{N}-{\bar{I}}_{male}{}^{O}<0$. Of these, 51% of the gap was within occupational categories, and 49% of the gap was between occupational categories. The income equation coefficient explains 37% of the income gap (Table 5 Panel B Part (1)), while the independent variable explains 15% of the gap (Table 5 Panel B Part (2)). Coefficient differences, or weight premiums, are the main cause of the income gap within occupational categories. Education and other characteristic variables caused differences in occupational structure, explaining 43% of the earnings gap (Table 5 Panel B Part (3)), while feedback disparities in employment opportunities explained 18%. Overweight men have higher income largely attributable to the weight premium.

Panel C in Table 5 shows the income gap decomposition of normal weight and underweight men. The income gap between two groups is 0.21. The percentage of the income gap explained by the coefficient on body size is small, while the variations in characteristic variables between normal weight and underweight men explain most of the income gap with a contribution of 79% (Table 5 Panel C Part (2)). The leanness penalty explains only 11% of the income gap (Table 5 Panel C Part (1)).

### 3.5. Path 1: Body Shape–Health–Productivity

The income gap depends heavily on health. Self-rated health status (1–5 from very unhealthy to very healthy) and frequency of health impacting life (1–5 from always to never) were used as proxy variables for health. Their ordered logit regression results with body shape are reported in Table 6. Both male and female Model (8) showed that being overweight and underweight was detrimental to health status. Model (9) also indicates that being underweight can significantly affect life. 

We further included health status as an independent variable to verify the effect of workers’ health status on income. The results are shown in Table 7, where the coefficient of health status is positive. Regardless of gender, health status is significantly and positively related to workers’ income.

### 3.6. Path 2: Body Shape–Social-Earnings

Usually, increased social frequency improves one’s social capital and results in higher income. The results of the regression between body shape and socialization are shown in Table 8. Neighborhood social frequency and friend social frequency were used as proxy variables for socialization (1–7 frequency was increasing).

- In the female sample in Table 8 Panel A, the coefficient for overweight was positive, while the coefficient for underweight was negative. This indicates that overweight women are more likely to have high-frequency neighborhood socialization, while slim individuals are more likely to have low-frequency neighborhood socialization. Body shape had no significant effect on women’s socialization with friends;
- We further analyzed the effect of neighborhood socialization on income, and the female Model (14) results in Table 9 show that neighborhood socialization has a negative effect on income. Although overweight has a positive impact on neighborhood socialization for females, there is ultimately a negative effect on income, which is the obesity penalty;
- The results were reversed for the male sample in Table 8 Panel B: body shape had no significant effect on neighborhood socialization for men but a significant effect on friend socialization;
- Further analyzing the effect of friend socialization on income, the male Model (18) in Table 9 shows that friend socialization among men contributes to income growth. Therefore, the final result for the male sample is a leanness penalty.

## 4. Discussion

### 4.1. Discussion of Results

Differences in the income gap caused by body size have been observed between male and female workers. However, this has not been explored across gender by occupational group in China’s population. In this study, we confirm the gender differences in body shape effects on income and argue that body shape effects vary with occupation. 

In female services, sales, and clerks, where professional, intellectual, and technical content are less demanding, women with a slimmer body may be more desirable to employers and customers and have higher income levels. In contrast, overweight women suffer significant employment discrimination and wage penalties among managers, professionals, and technicians. Obesity is often perceived as a synonym for indiscipline, laziness, and lack of self-control. Higher paying jobs tend to require higher levels of competence and self-control, resulting in greater marginal variation in the obesity penalty. The conclusion that obese women suffer a wage penalty has been repeatedly confirmed [49,54]. Compared with obese men, most obese women also need to consider the issue of fertility, and the decline in labor efficiency after giving birth is another important factor affecting employment [55]. In blue-collar occupations, underweight workers are subconsciously perceived by employers as less productive and having poor health, while employers prefer more muscular and overweight men. In occupations that emphasize physical labor, overweight men suffer a nonsignificant wage penalty.

The decomposition results suggest that body shape has strong explanatory power for the income gap (Table 5 Part (1)) and has an impact on occupational structure (Table 5 Part (4)). However, men and women face different reinforcement effects: women face an obesity penalty reinforced with higher occupational rank; men face a thinness penalty that is reinforced with lower occupational rank. The literature describes the effect of men as a weight premium and the effect received by women as a weight penalty [7,37,46]. This is most likely caused by gender differences in occupational structure. We compared a greater proportion of women working in services, sales, and as clerks, and a larger proportion of men working in blue-collar jobs. Services, sales, and clerks’ jobs require many interactions with people, and the obesity penalty is enhanced with higher occupational levels. A much higher percentage of the male population is blue-collar, where high weight is often associated with strong, muscular males, while the leanness penalty is more pronounced in low ISEI occupations. Managers, professionals, and technicians rely more on expertise and intelligence, and the thinness penalty is weaker.

The premium and penalty of body shape affect earnings mainly through two paths: body shape is often associated with health status and social frequency, affecting productivity. Health capital is the major component of human capital that affects the competitiveness of workers in the labor market. Workers with healthy capital tend to obtain better employment opportunities. Employers choose healthy employees, and poor health leads to lower productivity. Overweight and underweight often correlates to low physical health and an increased risk of diseases [56,57,58], for which employers have to bear higher health insurance costs and lost production. However, in blue-collar jobs for men, being overweight is often associated with muscle. Blue-collar jobs require more strength than underweight males normally have [15]. 

In our regression results, overweight men were more likely to prefer high-frequency friend socialization, but underweight men were more likely to prefer low-frequency friend socialization. One possible explanation is that exercise is an essential method of socialization with friends, especially for men. An increase in sports among overweight men would increase opportunities to socialize with friends. 

### 4.2. Limitations and Future Directions

This paper only used BMI as a proxy variable for body shape, but there are other body shape variables that we have not tried, such as body fat [31,56,59] and ABSI (A Body Shape Index), which is a limitation of this paper. BMI is becoming an outdated index and other indexes such ABSI are replacing it over the spectrum of different medical fields [60,61,62,63]. The follow-up work will use more scientific indices to verify the conclusions. 

Secondly, the variable we used in our discussion of health pathways was self-assessed health status. Self-perception of health is influenced by other elements. Some studies show a social gradient in health as a function of a person’s occupation [44,64]. Less advantaged social groups report worse health status. Likewise, incorporation into the world of work has had a positive influence on women’s health. We did not take into account that these factors may bias the regression results. Further research will to consider the indigeneity and changes of health in the future.

## 5. Conclusions

To further verify the robustness of the findings in this paper, we performed the following tests. (1) After redefining overweight and underweight with the 20 and 80% percentiles, most of the results and the significance did not change greatly; (2) After replacing individual income with respondent’s labor income, the findings remained robust.

This paper argues through extensive empirical analysis that (1) women’s BMI negatively effects income and that the negative effect is reinforced as occupational rank increases. The obesity penalty is more pronounced in occupations with high ISEI; (2) men’s BMI positively effects income, and low weight is detrimental to higher income, that is, the thinness penalty. The thinness penalty for men is strongest in occupations with low ISEI, especially in blue-collar jobs with a large proportion of men; (3) after income gap decomposition, it was found that the obesity penalty explained 29% of the income loss of overweight women. The thinness penalty explained 11% of the income loss for thin males. The weight premium for males explained 37% of the income gap between overweight and normal weight males; (4) men and women face different body shape–income effects because of different occupational structures. Women have a greater share of workers in services, sales, and clerks, while men have a greater share of blue-collar occupations (craft, machine operators, and elementary occupations); (5) body shape affects health status, socialization, and ultimately productivity and income. 

This paper provides additional constructive research on the income gap and gender disparity. We analyze the income effect of body shape in terms of occupational structure, which broadens the research approach to labor market discrimination. It also provides more empirical evidence to investigate the income gap and body shape discrimination in the nonagricultural labor market in China. In order to ameliorate the negative effects of inequality brought about by body shape discrimination, the measures that can be considered include two aspects: on the one hand, it is necessary to improve labor laws, eliminate body discrimination in the labor market, establish scientific concepts of employment through policy guidance, and bring the maximum value of human capital. On the other hand, equal employment structure for men and women should be promoted. Government should take measures to promote women’s human capital, and higher levels of education and occupation would enable women less from body size discrimination.

## Figures and Tables

**Table 1:** Key variable description.

Variable Type	Variable	Variable Description
Income	Income	Logarithm of respondents’ previous year’s income
	Labor income	Logarithm of respondents’ labor income in previous year
Body shape	Body weight	Kilograms
	Height	Meters
	BMI	Body weight/height ^2^
	Overweight	BMI quartile in the top 30%
	Normal	BMI quartile between 31–70%
	Underweight/Slim	BMI quartile in the low 30%
Occupation	Occupation	1 = Managers 2 = Professionals 3 = Technicians and associate professionals 4 = Clerical support workers 5 = Services and sales workers 7 = Craft and related trades workers 8 = Plant and machine operators 9 = Elementary occupations
	ISEI	International socioeconomic index
Demographics	Education	1 = No education 2 = Primary school or below 3 = Junior high school 4 = High school and technical secondary school 5 = Junior college and undergraduate and above
	Migration	0 = Local hukou; 1 = Migrant hukou
	Race	0 = Others; 1 = Han
	Marital Status	0 = Single (unmarried, divorced or widowed) 1 = Married
	Children	Number of children under 18 years old
	Age	Logarithm of age
Socioeconomic status	Union	0 = not union member; 1 = union member
	Political status	0 = Non-Chinese Communist; 1 = Chinese Communist
	Medical insurance	0 = no; 1 = yes
	Social status	Social status of self-assessment 1–10
Region controls	PGDP	Logarithm of per capita gross regional product
	Population	Logarithm of resident population
	Number of unemployed	Logarithm of number of urban registered unemployed
	Number of benefits	Logarithm of number of people on unemployment benefits
	Consumption	Logarithm of consumption per capita
	Health institutions	Logarithm of number of medical and health institutions
	Hospitals	Logarithm of number of hospitals
	Health Technicians	Logarithm of number of medical and health technicians
Fixed effects	Area	i.area (East, Central, West)
	Year	i.year (2010, 2011, 2012, 2013, 2015 and 2017)
Other variables	Health	1 = Very unhealthy; 2 = Relatively unhealthy; 3 = Normal; 4 = Relatively healthy; 5 = Very healthy
	Health impact on life	1 = Always; 2 = Often; 3 = Sometimes; 4 = Rarely; 5 = Never
	Neighborhood social	1 = Never; 2 = Once a year or less; 3 = A few times a year; 4 = About once a month; 5 = A few times a month; 6 = 1 or 2 times a week; 7 = Almost every day;
	Friends social	

**Table 2:** Mean of key variables and occupational structure.

	Female				Male			
	All	Overweight	Normal	Underweight	All	Overweight	Normal	Underweight
Income	32,876	28,830	34,947	33,847	45,967	52,275	46,911	37,937
Logarithm of income	9.94	9.86	10.01	9.93	10.25	10.37	10.28	10.07
Height	1.60	1.60	1.60	1.61	1.71	1.71	1.71	1.71
Weight	55.34	66.47	55.69	43.51	66.16	79.48	66.71	51.66
BMI	21.57	26.07	21.67	16.84	22.59	27.07	22.81	17.64
ISEI	38.15	36.20	38.55	39.52	40.61	41.93	40.41	39.52
Occupations (%)								
Managers	4.85	4.50	5.20	4.68	8.97	11.10	8.65	7.20
Professionals	11.71	8.24	12.29	14.35	8.73	8.87	8.78	8.51
Technicians and associate professionals	9.68	8.70	9.19	11.41	9.97	10.94	9.67	9.38
Clerical support workers	12.25	11.11	12.77	12.61	6.77	7.56	7.08	5.54
Services and sales workers	32.94	32.88	33.15	32.67	20.80	21.17	20.42	20.94
Craft and related trades workers	10.95	12.36	10.65	9.97	20.02	16.28	20.40	23.36
Plant and machine operators and assemblers	7.40	9.61	7.14	5.56	14.17	15.20	13.56	13.96
Elementary occupations	10.22	12.60	9.61	8.74	10.57	8.89	11.45	11.10
Total	100	100	100	100	100	100	100	100

**Table 3:** Regression results of BMI: the pros and cons of income.

D.V.	Income				
	OLS	OLS	OLS	Heckman	2SLS
	Model (1)	Model (2)	Model (3)	Model (4)	Model (5)
	Panel A Female				
BMI	−0.028 ***	−0.010 ***	−0.010 ***	−0.010 ***	−0.151 ***
	(0.003)	(0.003)	(0.003)	(0.002)	(0.024)
Demographics		Yes	Yes	Yes	Yes
Socioeconomic status		Yes	Yes	Yes	Yes
Regional controls			Yes	Yes	Yes
Fixed effects	Yes	Yes	Yes	Yes	Yes
Observations	11,464	11,212	11,206	14,097	11,206
Adjusted R^2^	0.161	0.331	0.373		0.186
	Panel B Male				
BMI	0.022 ***	0.014 ***	0.017 ***	0.017 ***	0.058 ***
	(0.002)	(0.002)	(0.002)	(0.002)	(0.020)
Demographics		Yes	Yes	Yes	Yes
Socioeconomic status		Yes	Yes	Yes	Yes
Regional control			Yes	Yes	Yes
Fixed effects	Yes	Yes	Yes	Yes	Yes
Observations	14,125	13,851	13,841	15,336	13,841
Adjusted R^2^	0.138	0.310	0.347		0.330

Notes: (1) Robust standard in parentheses; (2) *** *p* < 0.01, ** *p* < 0.05, * *p* < 0.1. The same below. (3) For demographics, socioeconomic status, regional controls and fixed effects variables and their descriptions, please refer to Table 1.

**Table 4:** Regression results of the interaction effect between BMI and occupation.

D.V.	Income			
	Panel A Female		Panel B Male	
	Model (6)	Model (7)	Model (6)	Model (7)
BMI	−0.008 ***	−0.008 ***	0.005 ***	0.016 ***
	(0.002)	(0.003)	(0.002)	(0.002)
ISEI	0.022 ***	0.021 ***	0.018 ***	0.016 ***
	(0.003)	(0.003)	(0.003)	(0.003)
BMI*ISEI	−0.00052 ***	−0.00046 ***	−0.00027 **	−0.00019
	(0.000)	(0.000)	(0.000)	(0.000)
Demographics	Yes	Yes	Yes	Yes
Socioeconomic status	Yes	Yes	Yes	Yes
Regional control		Yes		Yes
Fixed effects		Yes		Yes
Observations	11,212	11,206	13,851	13,841
Adjusted R^2^	0.290	0.395	0.270	0.370

Notes: (1) Robust standard in parentheses; (2) *** *p* < 0.01, ** *p* < 0.05, * *p* < 0.1. The same below. (3) For demographics, socioeconomic status, regional controls and fixed effects variables and their descriptions, please refer to Table 1.

**Table 5:** Income gap decomposition.

	Part (1)	Part (2)	Part (4)	Part (5)	Total
Panel A Normal weight female—Overweight female					
Managers	0.005	0.001	0.021	0.047	0.074
Professionals	0.010	−0.001	0.341	0.151	0.500
Technicians and associate professionals	0.006	0.000	0.121	−0.116	0.011
Clerical support workers	0.008	0.012	0.172	0.020	0.212
Services and sales workers	0.016	0.026	0.009	0.081	0.132
Craft and related trades workers	0.004	0.002	−0.176	−0.067	−0.237
Plant and machine operators and assemblers	−0.006	0.004	−0.147	−0.081	−0.230
Elementary occupations	0.000	−0.006	−0.286	−0.025	−0.318
Total	0.042	0.039	0.054	0.011	0.145
Percentage	28.75%	26.71%	37.26%	7.28%	
Panel B Normal weight male—Overweight male					
Managers	−0.009	0.004	−0.048	−0.236	−0.290
Professionals	−0.003	−0.007	−0.082	0.060	−0.033
Technicians and associate professionals	−0.004	0.005	−0.075	−0.060	−0.134
Clerical support workers	0.006	0.003	−0.095	0.060	−0.026
Services and sales workers	−0.014	−0.001	0.059	−0.111	−0.066
Craft and related trades workers	−0.002	−0.006	0.094	0.324	0.411
Plant and machine operators and assemblers	−0.008	−0.007	0.003	−0.187	−0.199
Elementary occupations	0.000	−0.004	0.104	0.145	0.245
Total	−0.034	−0.014	−0.039	−0.005	−0.092
Percentage	36.79%	14.77%	42.84%	5.60%	
Panel C Normal weight male—Underweight male					
Managers	0.005	0.011	−0.503	0.616	0.129
Professionals	−0.006	0.016	0.313	−0.299	0.024
Technicians and associate professionals	0.003	0.017	0.300	−0.247	0.072
Clerical support workers	0.001	0.007	0.252	−0.071	0.188
Services and sales workers	0.018	0.035	0.408	−0.407	0.054
Craft and related trades workers	0.017	0.032	0.395	−0.729	−0.285
Plant and machine operators and assemblers	−0.017	0.036	−1.397	1.347	−0.032
Elementary occupations	0.002	0.014	0.229	−0.186	0.059
Total	0.023	0.167	−0.003	0.023	0.210
Percentage	11.02%	79.47%	−1.25%	10.76%	

**Table 6:** Ordered logit results of body shape impact on health status.

D.V.	Panel A Female		Panel B Male	
	Health	Health Impact on Life	Health	Health Impact on Life
	Model (8)	Model (9)	Model (8)	Model (9)
Overweight	−0.198 ***	−0.043	−0.171 ***	0.035
	(0.038)	(0.040)	(0.036)	(0.038)
Underweight	−0.106 **	−0.135 ***	−0.165 ***	−0.147 ***
	(0.041)	(0.043)	(0.040)	(0.042)
Demographics	Yes	Yes	Yes	Yes
Socioeconomic status	Yes	Yes	Yes	Yes
Regional control	Yes	Yes	Yes	Yes
Fixed effects	Yes	Yes	Yes	Yes
Observations	14,089	14,063	15,326	15,301
Pseudo R^2^	0.054	0.110	0.061	0.125

Notes: (1) Robust standard in parentheses; (2) *** *p* < 0.01, ** *p* < 0.05, * *p* < 0.1. The same below. (3) For demographics, socioeconomic status, regional controls and fixed effects variables and their descriptions, please refer to Table 1.

**Table 7:** Regression results of the health impact on income.

D.V.	Income			
	Panel A Female		Panel B Male	
	Model (10)	Model (11)	Model (10)	Model (11)
Health	0.061 ***		0.073 ***	
	(0.008)		(0.008)	
Health impact on life		0.049 ***		0.091 ***
		(0.009)		(0.008)
Demographics	Yes	Yes	Yes	Yes
Socioeconomic status	Yes	Yes	Yes	Yes
Regional control	Yes	Yes	Yes	Yes
Fixed effects	Yes	Yes	Yes	Yes
Observations	11,200	11,181	13,832	13,810
Adjusted R^2^	0.375	0.374	0.349	0.351

Notes: (1) Robust standard in parentheses; (2) *** *p* < 0.01, ** *p* < 0.05, * *p* < 0.1. The same below. (3) For demographics, socioeconomic status, regional controls and fixed effects variables and their descriptions, please refer to Table 1.

**Table 8:** Ordered logit results of body shape impact on socialization.

D.V.	Panel A Female		Panel B Male	
	Neighborhood Social	Friends Social	Neighborhood Social	Friends Social
	Model (12)	Model (13)	Model (12)	Model (13)
Overweight	0.077 *	−0.066	−0.014	0.068 *
	(0.042)	(0.043)	(0.039)	(0.039)
Underweight	−0.091 **	0.040	0.017	−0.112 ***
	(0.045)	(0.043)	(0.043)	(0.043)
Demographics	Yes	Yes	Yes	Yes
Socioeconomic status	Yes	Yes	Yes	Yes
Regional control	Yes	Yes	Yes	Yes
Fixed effects	Yes	Yes	Yes	Yes
Observations	11,176	11,174	12,238	12,238
Pseudo R^2^	0.038	0.019	0.040	0.024

Notes: (1) Robust standard in parentheses; (2) *** *p* < 0.01, ** *p* < 0.05, * *p* < 0.1. The same below. (3) For demographics, socioeconomic status, regional controls and fixed effects variables and their descriptions, please refer to Table 1.

**Table 9:** Regression results of socialization impact on income.

D.V.	Income	
	Female	Male
	Model (14)	Model (15)
Neighborhood social	−0.035 ***	
	(0.004)	
Friends social		0.053 ***
		(0.005)
Demographics	Yes	Yes
Socioeconomic status	Yes	Yes
Regional control	Yes	Yes
Fixed effects	Yes	Yes
Observations	8991	11,145
Adjusted R^2^	0.364	0.339

Notes: (1) Robust standard in parentheses; (2) *** *p* < 0.01, ** *p* < 0.05, * *p* < 0.1. The same below. (3) For demographics, socioeconomic status, regional controls and fixed effects variables and their descriptions, please refer to Table 1.

## Data Availability

CGSS data available at http://cgss.ruc.edu.cn/ (accessed on 9 November 2020).
